# A case report of breast cancer in silicone-injected breasts diagnosed by an emerging technique of contrast-enhanced mammography-guided biopsy

**DOI:** 10.3389/fonc.2022.884576

**Published:** 2022-07-22

**Authors:** Yun-Chung Cheung, Wen-Lin Kuo, Li-Yu Lee, Ya-Chun Tang

**Affiliations:** ^1^ Department of Medical Imaging and Intervention, Chang Gung Memorial Hospital, Medical College of Chang Gung University, Taoyuan, Taiwan; ^2^ Division of Breast Surgery, Department of Surgery, Chang Gung Memorial Hospital, Medical College of Chang Gung University, Taoyuan, Taiwan; ^3^ Department of Pathology, Chang Gung Memorial Hospital, Medical College of Chang Gung University, Taoyuan, Taiwan

**Keywords:** silicone-injected breast, breast cancer, contrast-enhanced spectral mammography, breast biopsy, contrast-enhanced mammography-guided biopsy

## Abstract

**Background:**

Breast cancer in silicone-injected breasts is often obscured in conventional mammography and sonography. Contrast-enhanced magnetic resonance imaging (CE-MRI) is an optimal modality for cancer detection. This case report demonstrates the use of contrast-enhanced spectral mammography (CESM) and CESM-guided biopsy (CESM-Bx) to diagnose breast cancer in silicone-injected breasts. However, there is no relevant report in the literature.

**Case Presentation:**

A 59-year-old woman who received a liquid silicone injection for breast augmentation 30 years ago was transferred to our hospital for a CE-MRI-guided biopsy due to a suspicion of cancer in her right breast. The CE-MRI showed a 3.1-cm irregular enhanced mass and a 1.1-cm circumscribe mass in the upper outer quadrant of the right breast. Unfortunately, the CE-MRI-guided biopsy had to wait for 1 month due to a busy schedule. The CESM revealed two masses that were consistent with CE-MRI findings. CESM-Bx was performed, and the patient was diagnosed with invasive lobular carcinoma with an irregular mass and fibroadenoma of the circumscribed mass. The patient underwent substantial surgery.

**Conclusions:**

CESM-Bx is a simple emerging technique that can be used feasibly to obtain tissue proof on the concerned enhanced lesion on CESM. In such cases of silicone-injected breasts, the CESM-Bx can be used as an alternative to MRI-guided biopsy for cancer diagnosis.

## Introduction

Liquid silicone injection for breast augmentation was initiated worldwide in the 1950s and 1960s (1). Although the United States Food and Drug Administration (US FDA) has never approved the use of silicone injection, it has been illicitly performed by physicians and non-physicians in the United States, Mexico, and Asia (2). Women who have received silicone injection experience a foreign body reaction associated with the clinical symptoms of breast masses or pain. The complications of inflammation or fibrosis influence the differentiation among the foreign body granulomas (also called siliconomas), inflammatory masses, mastitis, and breast cancer (3–5). That is the reason why the US FDA officially banned the use of all silicone injection products for medical procedures in 1982 (6).

Women who have received silicone injections for breast augmentation are now at the age of higher incidence of breast cancer. For conventional breast imaging examinations, the observation of masses is always interrupted by the dense parenchymal fibrosis on mammography or by the diffused strong acoustic shadowing on sonography (6). Currently, contrast-enhanced magnetic resonance imaging (CE-MRI) has been documented as the best modality for silicone-injected breasts. The enhancement technique can detect angiogenic lesions; unfortunately, the enhancement is often unable to characterize breast malignant tumors, mastitis, inflammatory mass, or angiogenic benign tumors. For suspicious enhanced lesions, CE-MRI-guided biopsy is a preferable solution for tissue proof (7, 8). However, the procedure cost, MRI machine availability, biopsy duration, and technical practicality are controversial for clinical application.

In this report, we introduce an emerging technique of contrast-enhanced spectral mammography-guided biopsy (CESM-Bx) to diagnose breast cancer in silicone-injected breasts. The imaging features of contrast-enhanced spectral mammography (CESM) and CE-MRI are presented. However, there are no relevant reports in the literature.

## Case description

### Patient information

**Figure 1:**
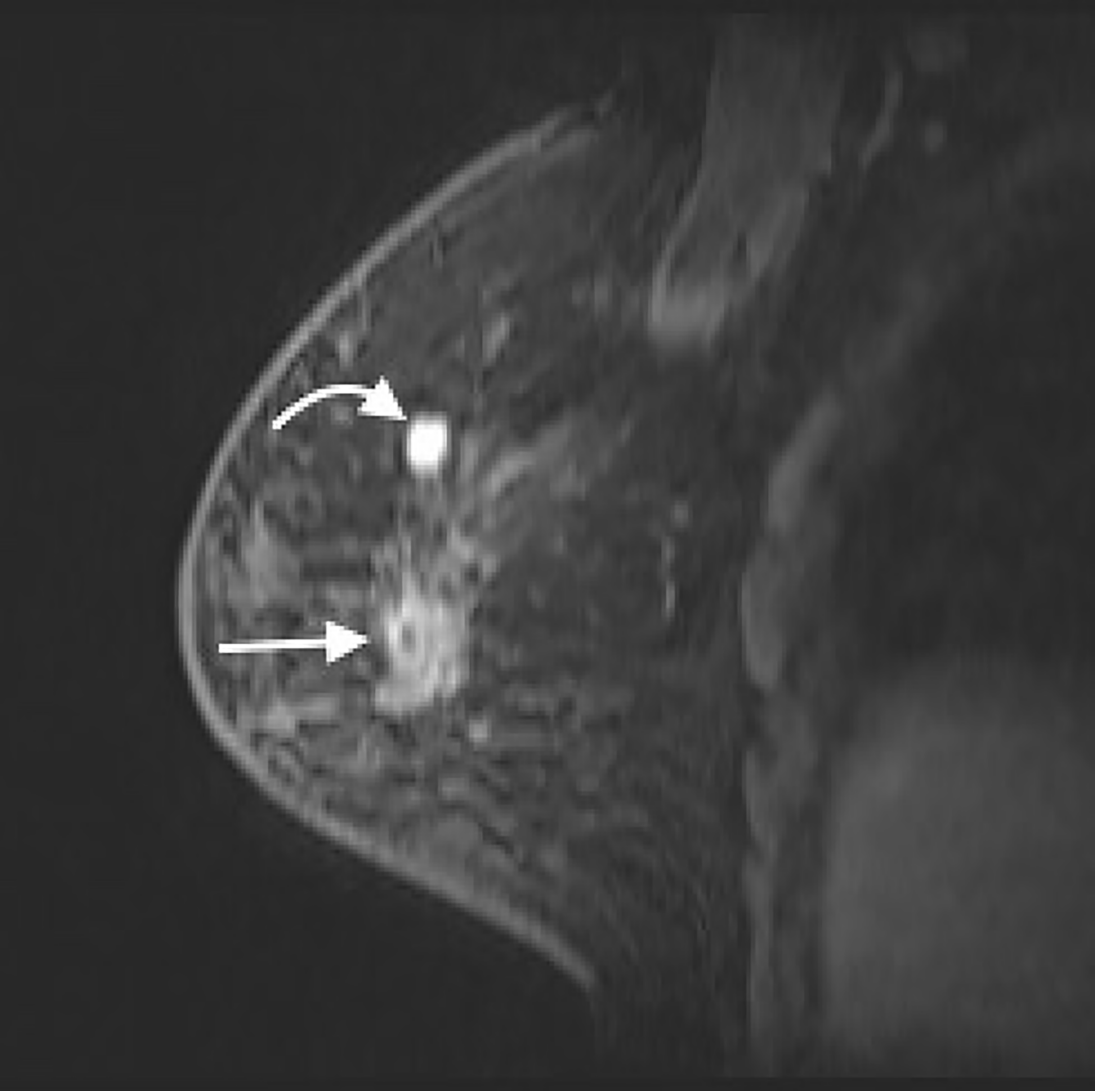
CE-MRI (sagittal view) of right breast showed a 3.1-cm irregular enhanced mass (straight arrow) and a 1.1-cm circumscribe enhanced mass (curved arrow) in the upper region of right breast. CE-MRI, contrast-enhanced magnetic resonance imaging.

A 59-year-old woman presented with a palpable mass with occasional pain in her right breast for 3 months. She had received a silicone oil injection in both breasts over 30 years ago. She subsequently underwent CE-MRI, which showed a 3.1-cm irregular enhanced mass and a 1.1-cm circumscribe enhanced mass in the upper outer quadrant of her right breast (**Figure 1**). The former mass revealed a persistent dynamic enhancement curve (type 1), and the latter was enhanced with a plateau (type 2). Silicone-induced inflammatory masses and breast cancer were also suspected. She was then transferred to our hospital for an MRI-guided biopsy.

Due to the tight schedule of MRI examinations in our hospital, the CE-MRI-guided biopsy needed to wait for a month. After a discussion with the patient, we decided to perform CESM to examine whether a new CESM-guided biopsy could be used as an alternative to an MRI-guided biopsy.

### Performance of contrast-enhanced spectral mammography and contrast-enhanced spectral mammography-guided biopsy

**Figure 2:**
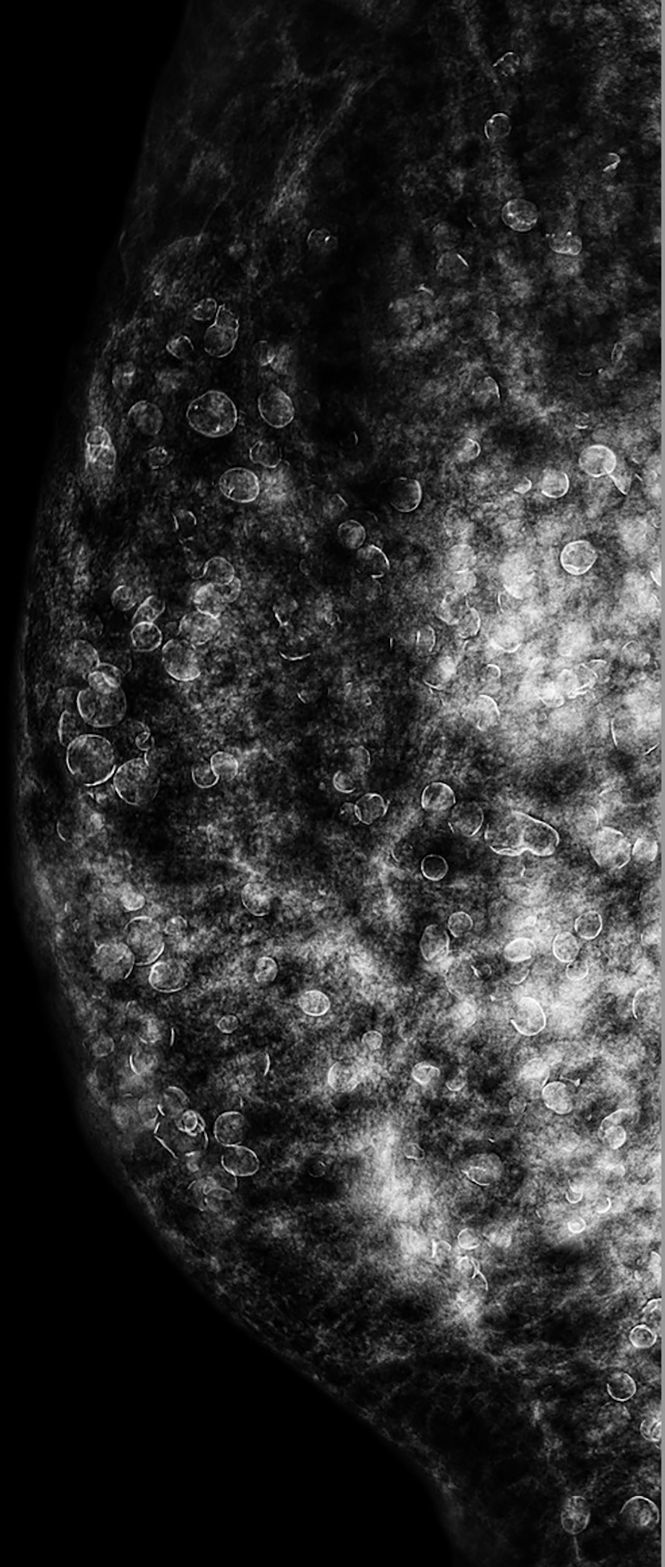
CESM (LM in lateral view) of the right breast showed inhomogeneous dense breast and numerous droplets of injected silicone with rim calcifications. The masses on CE-MRI were not observed. CESM, contrast-enhanced spectral mammography; LM, low-energy mammogram; CE-MRI, contrast-enhanced magnetic resonance imaging.

**Figure 3:**
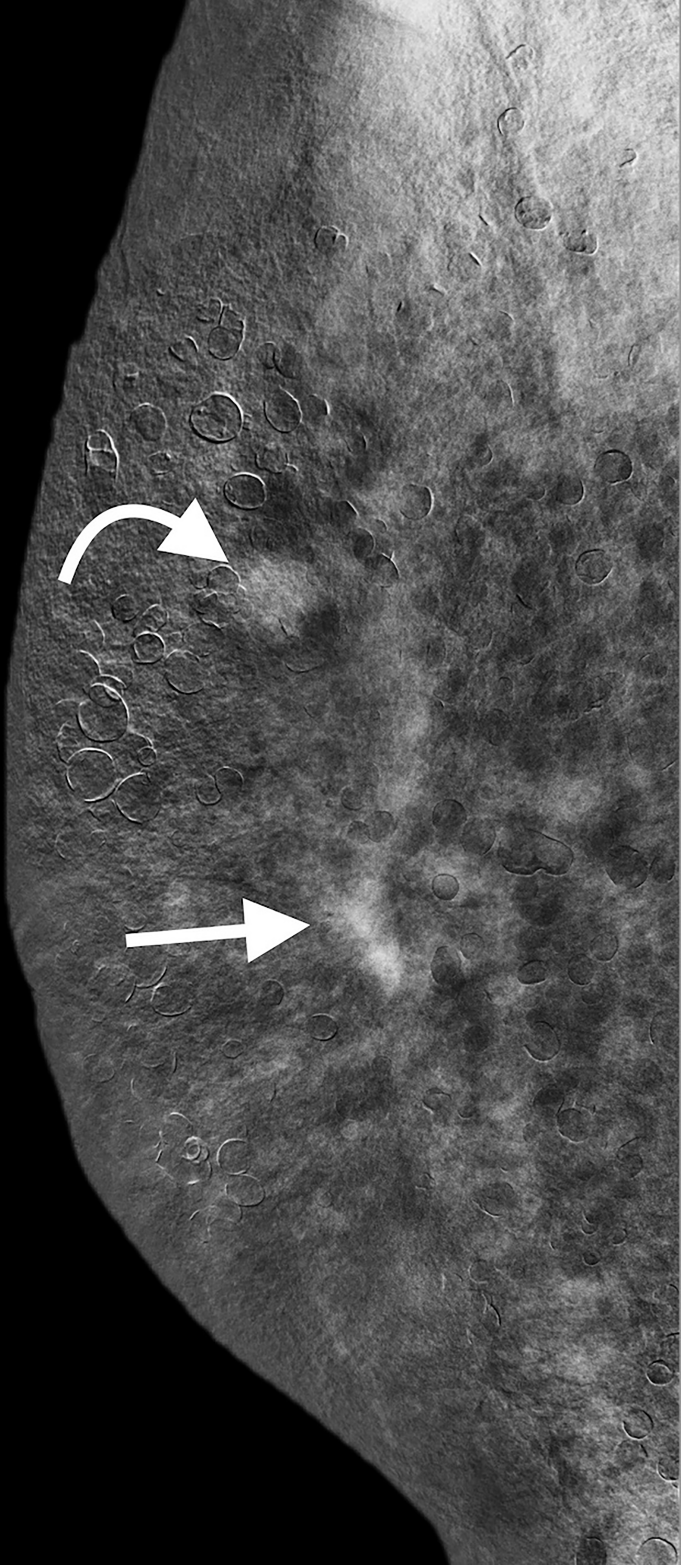
CESM (REI in lateral view) revealed the irregular enhanced mass (straight arrow) and circumscribe enhanced mass (curved arrow) in the right breast, which were compatible with CE-MRI findings. CESM, contrast-enhanced spectral mammography; REI, recombine enhanced image; CE-MRI, contrast-enhanced magnetic resonance imaging.

The CESM (Pristina; GE Healthcare, Buc, France) examination was routinely performed with intermittent exposure (approximately 2-s intervals) to low and high energy during a single breast-compressed position. The image acquisitions were obtained in the sequence of craniocaudal and lateral views of both breasts within 2–6 min after the start of a single bolus injection of non-ionic contrast medium (Omnipaque 350 mg I/ml; GE Healthcare, Dublin, Ireland) at a rate of 3 ml/s for a total dose of 1.5 ml/kg body weight *via* an intravenous catheter inserted into the forearm prior to the examination. In the same position of the breast, a low-energy mammogram (LM) and recombine enhanced image (REI) were provided for interpretation. LM showed numerous silicone droplets with rim calcifications diffusely in both breasts (**Figure 2**). The masses in the right breast could not be identified. However, the REI revealed an enhanced irregular mass and circumscribed mass in the right breast (**Figure 3**), which were compatible with the CE-MRI findings. CESM-Bx was then arranged a day after the CESM examination.

**Figure 4:**
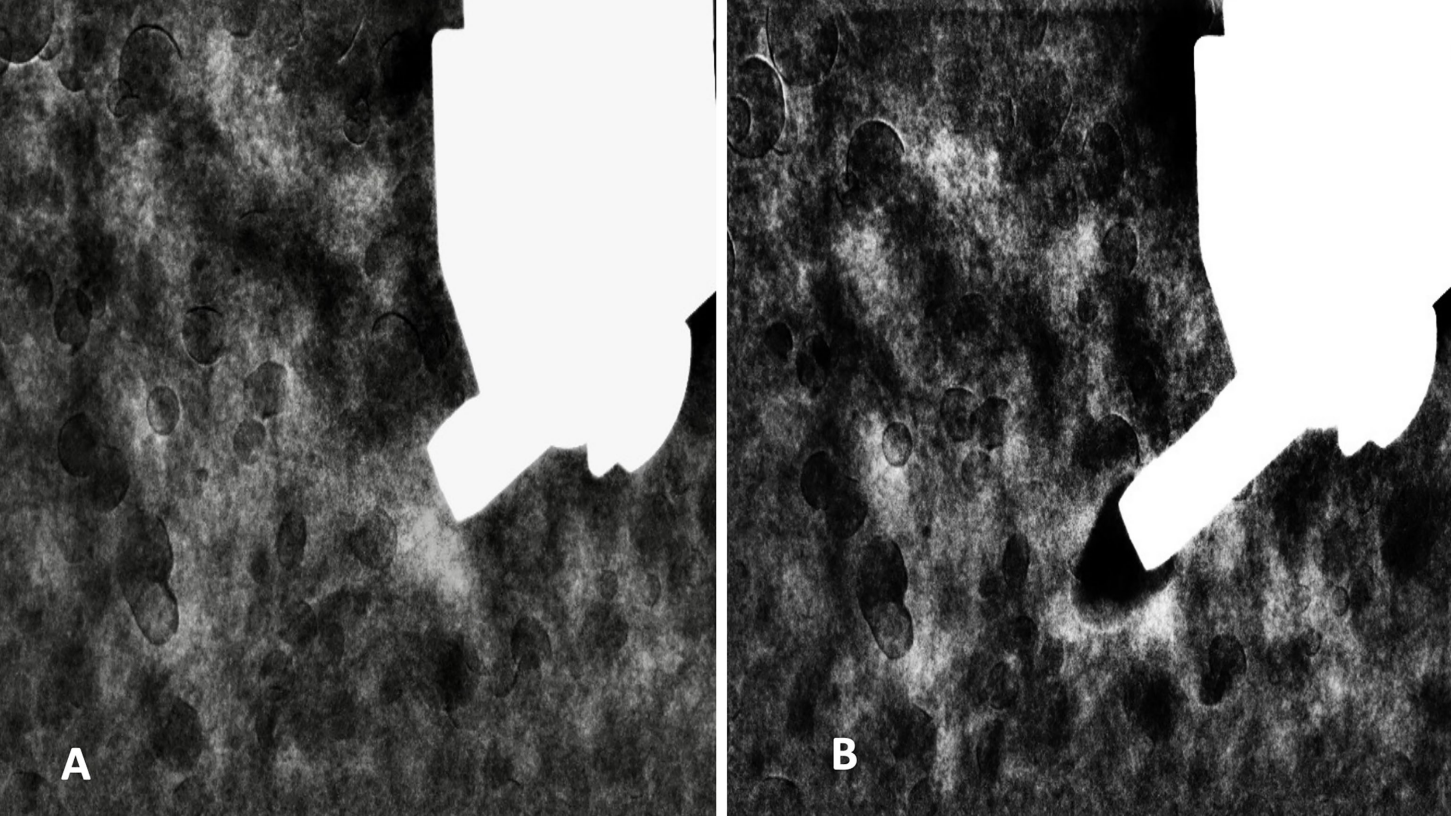
REI before **(A)** and after **(B)** CESM-Bx demonstrated successful biopsy of the enhanced mass. REI, recombine enhanced image; CESM-Bx, contrast-enhanced spectral mammography-guided biopsy.

In correlation to the CESM, we marked the approximate locations of the targets with a red pen on the skin. We utilized the same mammographic unit and injection protocol of contrast medium prior to the CESM-Bx. Vacuum-assisted biopsy (7-gauge biopsy needle with Encor biopsy system; Bard) was used with the patient lying in decubitus position, and the biopsy needle was set up in a vertical approach from lateral to medial into the breast. Two minutes after the start of contrast medium injection, the marked target was localized and compressed within the biopsy window. With stereotactic localization of the three imaging exposures at +15°, 0°, and −15°, the computer provided the co-ordinations of the chosen targets. After confirmation that the needle was in front of the target, it was fired through the lesion, and four multidirectional suction samplings were performed in complete clockwise allocation (**Figure 4**). The biopsy was performed on the two enhanced masses individually, and it took 9 min from the start of the contrast medium injection.

### Diagnostic assessment

The specimens from the two targets were separately sent for pathological study. Microscopic examinations revealed invasive lobular carcinoma (ILC) with an irregular enhanced mass and fibroadenoma for the circumscribed enhanced mass. The patient finally underwent a total mastectomy, confirming invasive lobular carcinoma and fibroadenoma.

## Discussion

Cancer in the augmented breasts has been reported with a rate ranging from 0.2% to 2.7% (9). However, most of the augmented patients in the series were treated with bag prosthesis or paraffin injection. The details for those following silicone injections were not thoroughly investigated. All kinds of malignant tumors can exist including the most common invasive ductal carcinoma and the uncommon cancers of adenocarcinoma or squamous cell carcinoma (10, 11). In a series of lumpy silicone-injected breasts, only three of 16 cases were histologically proven to be invasive ductal cancer. Most were silicone-related pathologies (12). Diagnosis of cancer in these silicone-injected breasts is thus important for treatment.

There is no evidence of silicone injection relating to cancer; however, the reactive changes in the breast induce difficulty in observing breast cancer by conventional mammography and sonography. The typical diffuse distribution of silicone granulomas or dense fibrosis on a mammogram can obscure the coexisting neoplasms (13). The loss of posterior parenchymal definition and diffuse acoustic shadowing due to strong reflection, refraction, reverberation, and overwhelming sonographic beam on sonography limits cancer detection (13). Currently, CE-MRI has been documented as the best modality for silicone-injected breasts (14, 15). The injection of gadolinium can enhance angiogenic breast lesions; unfortunately, it often reveals the overlap enhancement features among the inflammation, and malignant or benign tumors (12). A CE-MRI-guided biopsy is thus essentially needed for diagnosis.

CESM is a novel mammography-based imaging examination that has been approved for clinical use by the US FDA in 2011 (14). Utilizing the different attenuation coefficients of iodine and glandular tissues under low- and high-energy intermittent exposures, computer software recombines these two images after eliminating the breast tissue background. The highlight on REI represents the presence of iodine accumulation that indicates possible pathogenic lesions. The cancer sensitivity and specificity of CESM range from 93% to 100% and 63% to 88%, respectively, which were significantly improved compared to those of full-field digital mammography (15–17). The cancer sensitivity and specificity of dense breasts increased by approximately 22% and 16%, respectively (18). The sensitivity of CESM has been documented to be comparative to that of CE-MRI (19, 20), making approximate pre-operative detection of multifocal or multicentric cancers (21–23). Importantly, a systemic review and meta-analysis further reported that CESM has higher specificity, positive predictive value, and diagnostic confidence rate than MRI (24). However, CESM also has true positives and false positives resembling other modalities (25). CESM-Bx is therefore essentially approved by the US FDA in 2020 (26).

The ILC diagnosed in this case often presents synchronous multifocal, multicentric, or contralateral cancers in 20% to 29% (27). Unfortunately, the detection by mammography or physical examination is difficult because of the histologic features of single-file patterns and the lack of desmoplastic stroma reaction of cancer cells (27, 28). These problems of cancer detection or extent underestimation are associated with re-operation (29). The American College of Radiology (ACR) has recommended ILC as one of the indications for MRI examination due to the challenge of detection (30), in which the findings may change surgical planning. A series of ILC concluded that CESM could accurately assess the cancer extent with 0.87 Pearson’s correction. Only two (6.7%) of 30 cases were needed for re-excision (31). It seems that CESM and CESM-guided biopsy are meaningful for ILC assessment.

The CE-MRI-guided biopsy is the preferable procedure for histologic diagnosis in such difficult cases of percutaneous silicone-injected breasts. Unfortunately, it has several limitations including the examination cost, MRI machine availability, biopsy duration, and technical practicality. In our hospital, the examination fee for CE-MRI-guided biopsy is double or more than that of CESM-Bx. The heavy daily workload of MRI examinations always limits the machine’s availability. For this case report, the MRI-guided biopsy needed to wait for a month. Conversely, CESM-Bx is rather flexible with results obtained 2 days after CESM. For the procedure duration, the whole procedure of this CESM-Bx took 30 min, and the CE-MRI-guided biopsy took about 60 min. The CESM-Bx is easy in that the technique is approximately the same as the stereotactic mammography-guided biopsy, which is familiar to breast radiologists in their daily practice. Otherwise, the problem of a small breast or thin breast after compression, which often restricts the biopsy, can be solved by the horizontal approach of the biopsy needle.

## Conclusion

CESM-Bx is a simple and feasible technique for diagnosing concerned enhanced lesions on CESM. In the case of silicone-injected breasts, CESM-guided biopsy may be used as an alternative to CE-MRI-guided biopsy.

## Patient perspective

The patient appreciated the rapid arrangement of biopsy for treatment decision and the smooth performance of CESM-Bx.

## Data availability statement

The original contributions presented in the study are included in the article/supplementary material. Further inquiries can be directed to the corresponding author.

## Ethics statement

The studies involving human participants were reviewed and approved by Chang Gung Memorial Hospital IRB (No.: 202100839B0). The patients/participants provided their written informed consent to participate in this study.

## Author contributions

Y-CC made substantial contributions to the conception, design of the work, and data acquisition, analysis, and interpretation and drafted the work. W-LK made substantial contributions to patient treatment and care. L-YL made substantial contributions to the interpretation of pathology and substantially revised it. Y-CT made substantial contributions to the works on imaging and biopsy. All authors contributed to the article and approved the submitted version.

## Funding

This work was supported by the Chang Gung Memorial Hospital (Grant No. CMRPG3L1701).

## Conflict of interest

The authors declare that the research was conducted in the absence of any commercial or financial relationships that could be construed as a potential conflict of interest.

## Publisher’s note

All claims expressed in this article are solely those of the authors and do not necessarily represent those of their affiliated organizations, or those of the publisher, the editors and the reviewers. Any product that may be evaluated in this article, or claim that may be made by its manufacturer, is not guaranteed or endorsed by the publisher.
